# The influence of Strzelin Quarry Lakes on small reservoir retention resources in the regional catchments

**DOI:** 10.1038/s41598-022-18777-6

**Published:** 2022-08-27

**Authors:** Bartosz Jawecki

**Affiliations:** Department of Landscape Architecture, Wrocław University of Environmental and Life Sciences, ul. Grunwaldzka 55, 50-357 Wrocław, Poland

**Keywords:** Environmental sciences, Hydrology

## Abstract

The paper presents the results of the analysis of the volume of water retained in Strzelin Quarry Lakes (SQLs). The volume of retained water was estimated by using the computational method, where the proposed reduction factors were determined with the use of DTM (digital terrain model). 2.6 hm^3^ of water was retained in seventeen Strzelin Quarry Lakes, of which 1.2 hm^3^ in the Ślęza River catchment (3 quarry lakes), and 1.4 hm^3^ in the Oława River catchment (14 quarry lakes). The obtained data of the volume of water retained in SQLs were compared to the balance of the water retention capacity of water reservoirs in the catchments of the Ślęza River (0.809 hm^3^), part of the WR08 Bystrzyca balance catchments (16.190 hm^3^) and in the catchments of the Oława River (2.782 hm^3^), part of the WR09 Nysa Kłodzka balance catchment (104.960 hm^3^). Inclusion the volume of water retained in Strzelin Quarry Lakes in the small scale water retention (reservoirs and ponds) balance would increase the volume of retained water by 156.0% in the Ślęza catchment (by 7.8% in the WR08 Bystrzyca balance catchment) and by 49.5% in the Oława catchment (by 1,3% in the WR09 Nysa Kłodzka balance catchment). Under favorable hydrogeological and geomorphological conditions water reclamation of the excavations may be one of the main aspects of increasing the retention capacity of the catchment, what is particularly important in areas characterized by low water resources.

## Introduction

The total amount of freshwater resources in Europe is around 2270 km^3^/year^1^, while the average freshwater resources in Poland in the years 2000–2019 are approx. 57.7 km^3^/year^2^. According to estimations, European water resources amount, on average, to approx. 4.7 dam^3^/year per person and in Poland 1.4–1.5 dam^3^/year per person^2,3^. As Polish resources of water are considered to be one of the smallest in Europe, it is so important to properly shape and protect them against periodical water surplus or deficit, which is ensured, among others, by water retention in reservoirs^3^. Polish retention reservoirs are characterised by small capacity, which does not exceed 6.0% of the volume of the annual outflow of water from the territory of the country^2^. The total capacity of approx. 100 Polish retention reservoirs (of a unit total volume over 2 hm^3^) is approx. 3.5 km^3^^4^. Moreover, approx. 31.8 thousand small-scale retention objects (of a capacity up to 5 hm^3^^5–7^ may retain about 0.85 km^3^ of water^8^. At the same time, about 7 000 large reservoirs could be found across Europe, with a total capacity representing about 20.0% of the total freshwater resource^1^.

Small-scale retention is defined as all technical and non-technical solutions to improve the water balance of the catchment by increasing its natural and artificial retention capacity^9^. The natural forms of small scale retention are: landscaping in the catchment, an increase in soil retention and reduction of soil erosion, preservation and revitalization of hydrogenic habitats^5,9,10^. The technical forms of water retention include, first of all, water reservoirs of different sizes and purposes (ponds, field agricultural reservoirs, fire water reservoirs, oxbow lakes, moats, mine/quarry pits) and installations that enable water level adjustment (weirs, gates, barrages) as well as flood polders, inter-dike areas, canal and trench systems connecting the main river with oxbow lakes, properly functioning drainage systems^5,6,10–14^.

According to estimates, the global extent of land area impacted by mining and quarrying is approx. 421 thousand km^2^, in Europe, exclusive of Russia, the estimated area impacted by active mining is about 40 thousand km^2^^15^. Most of these areas are open-pit mines. In Poland, mining areas cover about 422 km^2^, of which open-pit mines occupy approx. 347 km^2^^8^. Part of this area includes basins of open excavation pits. After the end of exploitation, mining areas have to be reclaimed. Throughout the world, the main reclamation and management directions of post-mining areas are^16–20^: agriculture^21^, forestry^22^, nature conservation and natural habitats^23–26^, economic^27–31^; aquatic^14,31–35^ and leisure and tourism^36–41^. The water direction in the reclamation of post-mining areas is important from the point of view of sustainable water management, as it contributes to the creation of artificial lakes—mine or quarry lakes^14,27,32,33,42^. Hundreds of new mine or quarry lakes have been created all over the World, e.g. in the USA, Australia, China, Canada, Czech Republic, Italy, Germany, Poland, UK, France, Sweden^14,25,31,32,43^, in former excavations of clay, sand and gravel, in former stone quarries and in former open-cast coal and lignite mines, in former open-cast ore metal mines^14,31,32,42–46^ when the mining and quarrying was discontinued.

Mine or quarry lakes may occur in pits, which, after the end of dewatering, slowly fill with groundwater, rainfall, and surface runoff^32,34,46,47^. The mine or quarry lakes created in closed mines may have different depths, surface areas and volume, depending on the type of the excavated raw material and the mining technology^14,32,35,43–45,48–50^. Depending on the size of the open-cast mining pit, the hydrogeological conditions, and the geological structure of the mine and its surroundings, the mine flooding process may last for several months, up to several decades^14,32,33,45,49,51^. The potential use of mine and quarry lake water remains dependent on the water quantity and quality^35,43,44,48,52–54^. Mine or quarry lakes may be used for various purposes, among others: recreation and tourism^18,30,31,41,55^, wildlife habitats^16,18,26,27^, aquaculture and fish farming^18,40,43,56,57^, water management^14,16,31,40,43,56,58^ floating photovoltaic systems^59^, potable and industrial water reservoirs or irrigation water storage for agriculture and horticulture^31,40,43,45,52,53,55,57^, capturing flood waters, or improving the flow rate in water courses during droughts^42,43,60^.

Some important elements of Polish water management policy are adapting to climate changes, including counteracting the effects of floods and droughts (especially in the agriculture and water management sectors) and decreasing the water deficit^61–65^. Increasing the volume of retained water by means of creating various forms of water retention, including small-scale retention, is an important element of water management programmes and strategies^61–65^. It may also be one of the essential activities related to increasing water resources and their sustainable and rational management, especially in local and regional terms^3,9,66–71^. The increase in water retention in reservoirs may be complemented by water reclamation of closed pits that leads to the creation of mine or quarry lakes^14,27,33,38,43,56^. Mine and quarry lakes may be used, among others, to retain water or to capture flood waters and the water stored in them may serve as a source of potable or industrial water, for irrigation or aquaculture purposes and to increase the flows in water courses during periods of draught^31,33,42,43,45,52,53,57^ This is particularly important in areas characterised by low water resources^14,27,33,42,57^. However, mine and quarry lakes are not always taken into account in the reservoir retention balance; this applies particularly to small post-mining reservoirs^5,14,64,72^.

Studies and monitoring of mining and post-mining areas are commonly conducted with use of GIS and LiDAR technologies^14,32,73,74^. The morphological parameters of the pits, such as the capacity, basin surface, depth and water surface area^14,32,73,74^ may be determined with use of geodesic methods^75,76^, but for this purpose it is recommended to use LiDAR data obtained, among others, from terrestrial (TLS), airborne (ALS), and mobile (MLS) techniques or from other photogrammetric measurements, e.g. with use of Unmanned Aerial Vehicles (UAV)^14,32,76–82^. LiDAR data, digital terrain model (DTM) or digital elevation model (DEM), as well as orthophotomaps may also be used in water management, e.g. in forecasting the retention capacity of reservoirs and polders^14,32,83^, rainwater management^84–86^, modelling the risk of floods and their range^87–89^.

The aim of the present article is to use DTM (digital terrain model) to estimate the retention capacity of Strzelin Quarry Lakes and the influence of the volume of water retained in them on the volume of small-scale reservoir and pond retention resources in the catchments of the Ślęza (balance catchment W-VIII Bystrzyca) and Oława (balance catchment W-IX Nysa Kłodzka) Rivers, where the Strzelin quarries are located.

## Materials and methodology

**Figure 1:**
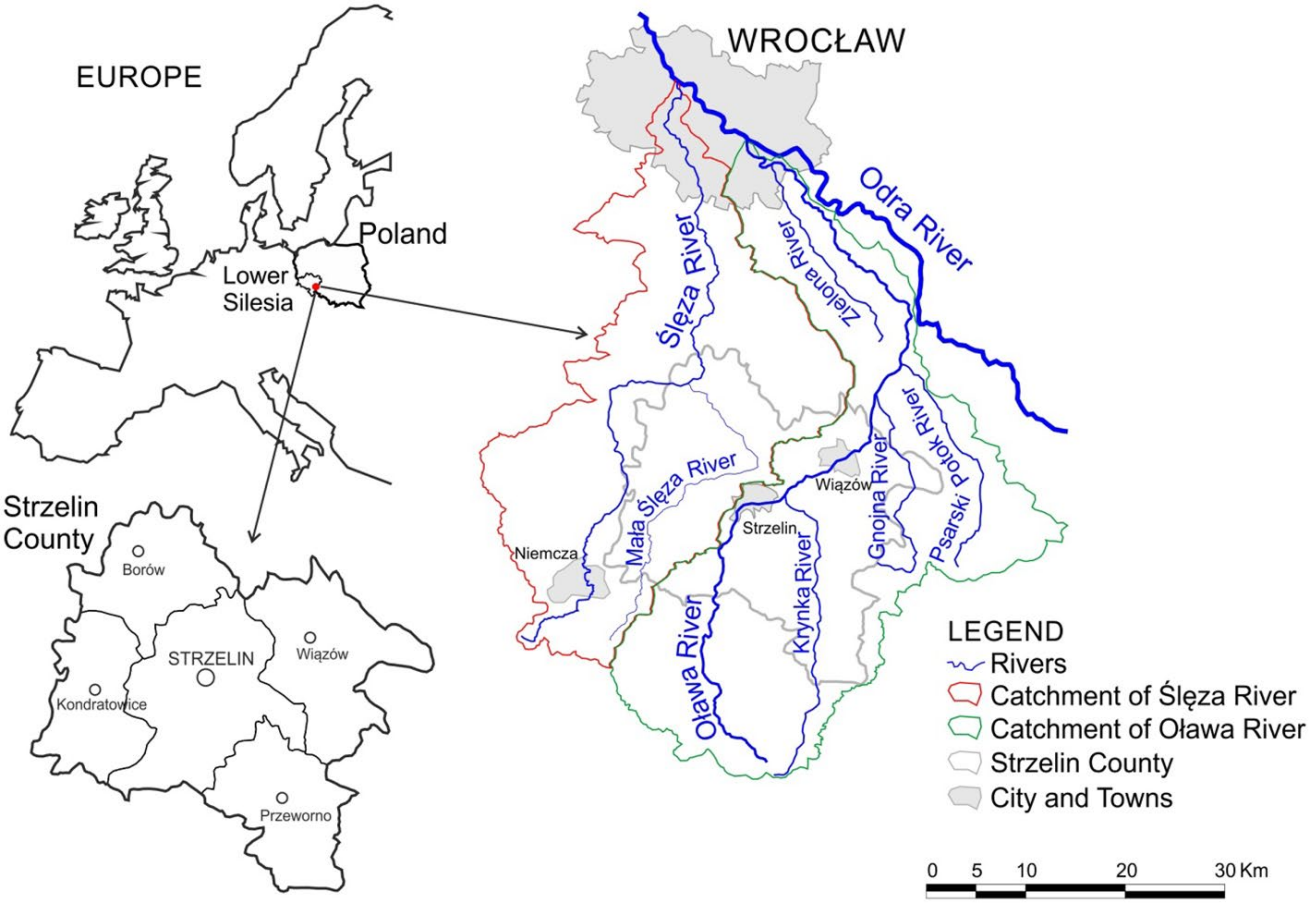
Catchments of the Ślęża and Oława Rivers in Europe and Poland.

The subject of the study are the Strzelin quarries situated in Lower Silesia (Poland, Central Europe) in Strzelin County, in the area of Strzelin Hills and Lipowe Hills^90–92^, in the catchments of the Oława and Ślęza Rivers, in the basin of the Oder River (Fig. 1)^93,94^. The name Strzelin Quarry Lakes (SQLs) refers to the flooded quarries of cohesive rock material located in Strzelin County.

**Figure 2:**
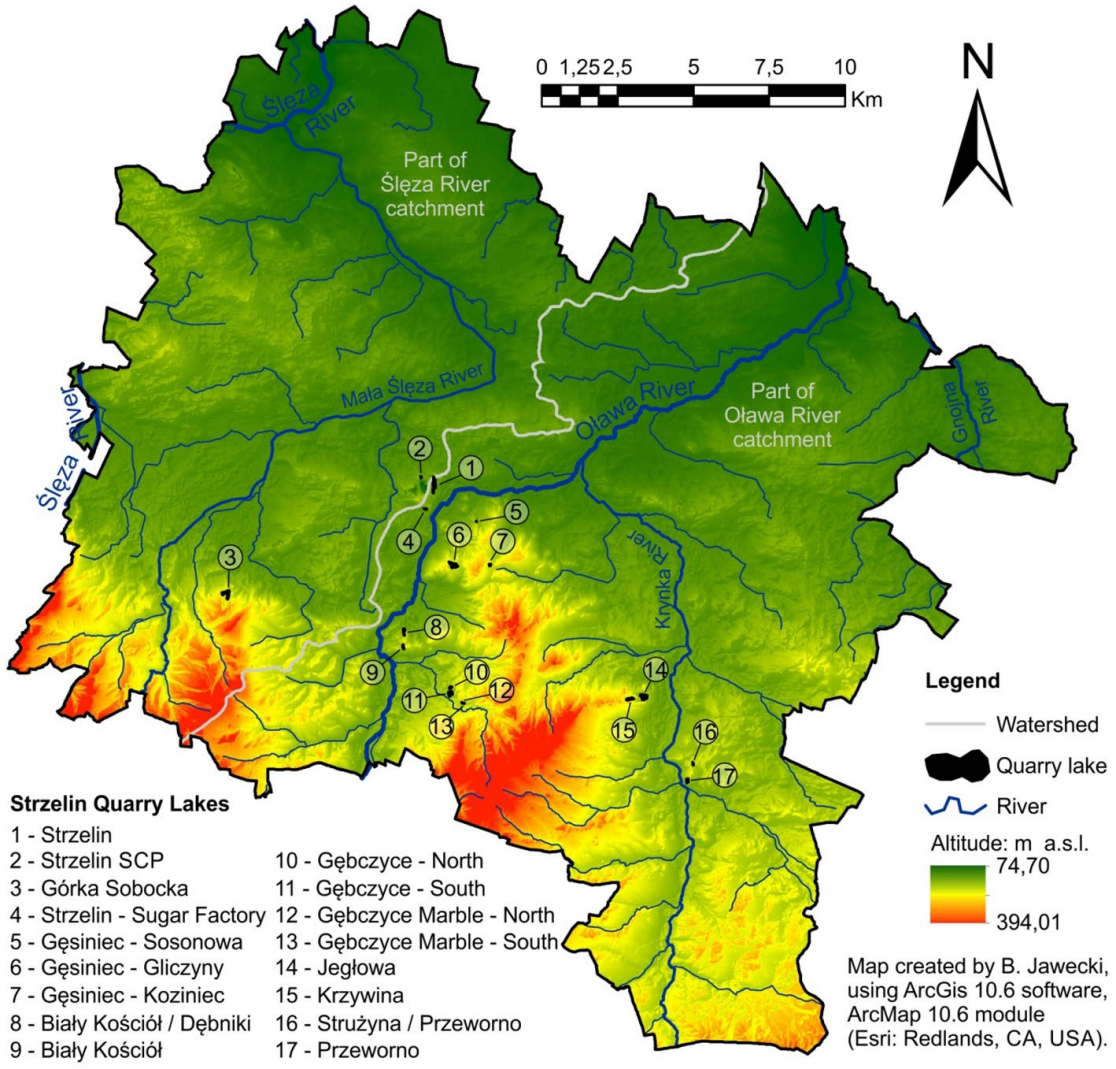
The location of Strzelin Quarry Lakes on the background of the hydrographic network of Strzelin County.

The Strzelin Hills consist of granitoides (granite, granodiorite, tonalite), gneiss, mica slates, quartzite, quartzite-sericite slate, amphibolite, calcareous/flint rocks, marble, and basalt, while Lipowe Hills contain gneiss, amphibolite, biotite-amphibolite slate, calcareous/flint rocks, granitoid, and basalt, partly covered by sedimentary rocks: silt, clay, and loess^28,95–98^. This fostered the exploitation of rock material, and, as a result, led to the presence of over 80 active or closed quarries of various sizes and depths. Quarry lakes have formed in some of them^14,32,52–54,95^. Figure 2 presents selected Strzelin Quarry Lakes on the background of the hydrographic network of Strzelin County.

The Oława River is a second rank water course of a length of 99.01 km and catchment area of 1134.4 km^2^. It is a left tributary of the Odra River, to which it flows in at km 250.4 in the city of Wrocław. Some of its main tributaries include Krynka, Gnojna, Psarski Potok, and Zielona. In the catchment, five consolidated, uniform water sections are distinguished. The catchment of Oława River constitutes a part of the balance catchment Nysa Kłodzka (W-IX), of a surface area of 4874.1 km^2^^94,99–102^. The Ślęza River is a second-rank water course of a length of 84.10 km and catchment area of 972.5 km^2^. It is a left tributary of the Odra River, to which it flows in at km 261.5 in the city of Wrocław, and its main tributaries are Krzywula, Oleszna, Kasina, Mała Ślęza, and Żurawka. In the catchment, 5 consolidated, uniform water sections are distinguished. The catchment of the Ślęza River constitutes part of the balance catchment Bystrzyca (W-VIII), of a surface area of 2753.8 km^2^^94,99–103^.

Based on the analyses of German topographic maps from the 1930s in a scale of 1:25,000^104,105^, contemporary topographic maps in the scale of 1:25,000 and 1:10,000 (license No. MGW.I.7522.524.2016_02_N), the Database of Topographic Objects—BDOT10k (licence no. MGW-I.7552.26.2019_02_N) orthophotomaps (licence no. DIO.7211.204.2019_PL_N) and the DTM of the Strzelin County that was created based on LiDAR ALS data (licence no. DIO.7211.160.2018_PL_N) created by author, with use of ArcGis software version 10.6, modules: ArcMap 10.6 & ArcScene 10.6 (Esri: Redlands, CA, USA), the quarry lakes in the Strzelin County were located. The water surface area of the analysed quarry lakes was determined with use of DTM (created in 2012) and orthophotomaps (created in 2016), by correlating both types of data in the ArcMap 10.6 environment^14,32^. On the other hand, during diving, the multi-gas manual diving computer SUUNTO VYTEC^53,54^ was used to determine the maximum depth of the reservoir in the flooded quarry. Tests were conducted on cohesive rock quarries, while other forms of mining and quarrying activities, e.g. sand and gravel quarry, clay quarry were omitted. The analyses were conducted on quarry lakes that retain water permanently, of a maximum depth exceeding 2 m, and a surface area over 1000 m^2^. Additionally, small, periodically drying ponds in close quarries and sumps in regularly drained active quarries were not analysed, either.

Due to the mining technology used in Strzelin quarries, it was assumed that their vertical structure was similar (steep vertical walls of the pit, and relatively flat bottom, transport ramps in smaller quarries), and that they varied in terms of the depth and surface area of the pits^14,26,95,106,107^.

The volume of water retained in quarry lakes was calculated as the product of multiplication of the surface area of the given reservoir and its average depth, with use of Eq. (1). The averaged depth of quarry lake was calculated based on the maximum depth of the quarry lake (D_max_) and the reduction factor (RF), with use of Eq. (2).

1 $$V=QLS \times {AD}_{q}$$

2 $${AD}_{q}= {D}_{max }\times RF$$

where: V is the estimated volume of water retained in quarry lake (m^3^), QLS is the area of water surface in the quarry lake (m^2^), AD_q_ is the average depth of quarry lake (m), D_max_ is the maximum depth of the quarry lake (m), RF is the reduction coefficient to reduce the maximum depth of the reservoir to the averaged value.

**Figure 3:**
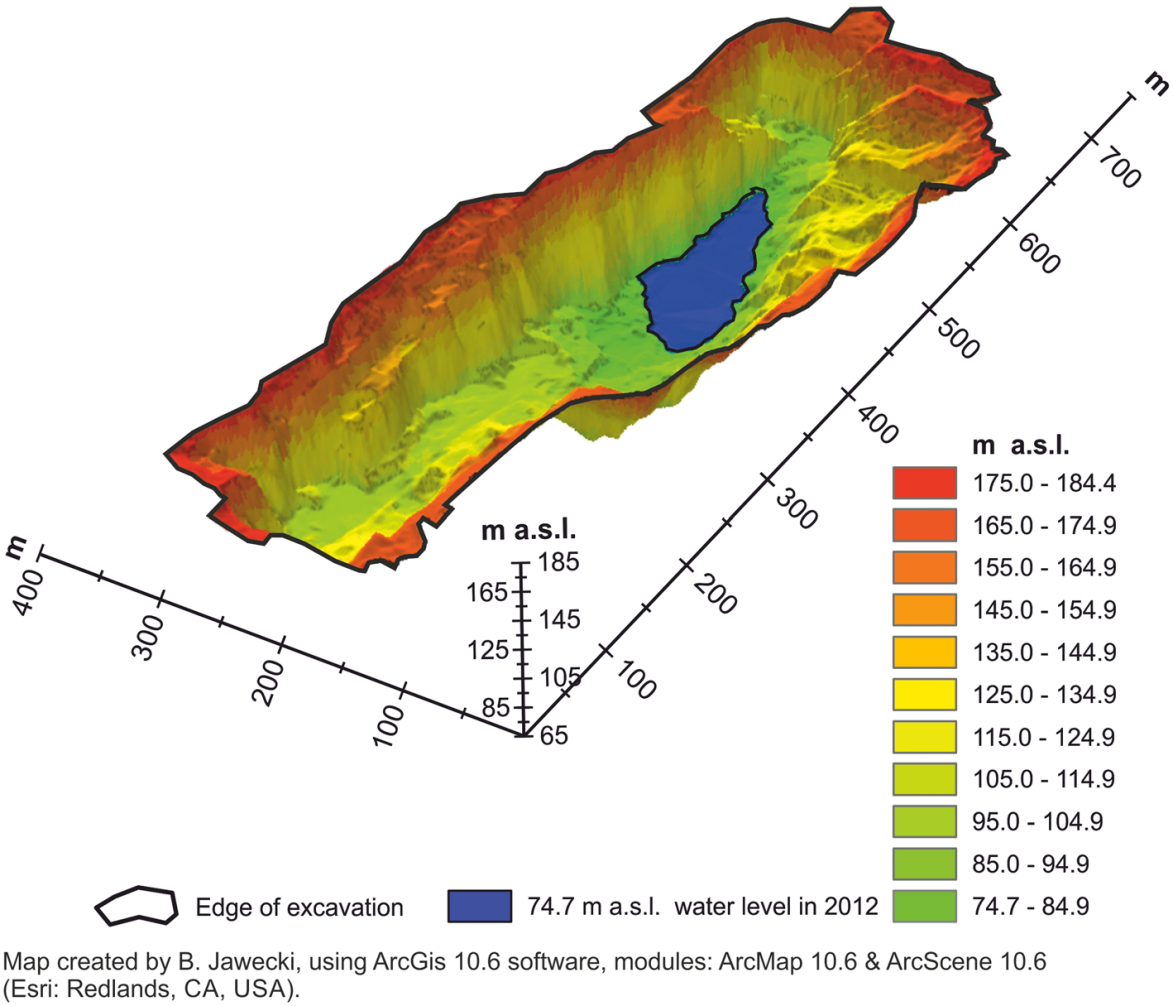
Digital Terrain Model of Strzelin Quarry, created based on LiDAR ALS data from 2012.

The RF coefficient was determined empirically based on DTM (Fig. 3) of the basin of quarry lake Strzelin^14,32^ that was created based on LiDAR ALS data collected on the 2012-04-27 as part of the ISOK (Informatyczny System Osłony Kraju, in English: Nation Protection IT System) project^14,32^. The averaged depth of the quarry lake Strzelin was calculated with use of Eq. (3) (calculations were performed for various stages of filling the pit with water (ordinates of the water level in the reservoir)) and Eq. (4). For each calculation instance, the individual reduction factor (IRF) was calculated from Eq. (5). The average reduction factor for actual values (ARF_n1, m1_) and the average reduction factor for actual and prognosed data (ARF_n2, m2_) were determined as the arithmetic mean value. Then, the RF reduction factor was calculated with use of Eq. (6). The calculations and values of the RF coefficient are presented in Table 1.

3 $${AD}_{n}= \frac{VRW}{SWR}$$

where: AD_n_ is the average depth (m), VRW is the volume of retained water (m^3^), SWR is the surface area of the water reservoir (m^2^).

4 $${AD}_{m}= \frac{VBO}{WSBO}$$

where: AD_m_ is the average depth (m), VBO is the volume of water between ordinates (m^3^), WSBO is the surface area of the water reservoir on the higher ordinate (m^2^).

5 $$IRF= \frac{{AD}_{n,m}}{{D}_{max}}$$

where: IRF is the individual reduction factor (–), AD_n,m_ is the average depth (m), D_max_ is the maximum depth of the water reservoir (m).

6 $$RF= \frac{{ARF}_{n1}+{ARF}_{n2}+{ARF}_{m1}+{ARF}_{m2}}{4}$$

where: RF is the reduction factor (–), ARF_n1,m1_ is the average reduction factor for actual data (–), ARF_n2,m2_ is the average reduction factor for actual and prognosed data (–).

**Table 1:** Determination of the RF reduction coefficient.

Ordinate of the water level	Volume of retained water ^a^ (VRW)	Surface area of the water reservoir (SWR)	Maximum depth of the water reservoir (D_max_)	Average depth (AD_n_)	Individual reduction factor (IRF)	Average reduction factor		Reduction factor (RF)
						(ARF_n1_)	(ARF_n2_)	
m a.s.l.	m^3^	m^2^	m	m	–	–	–	–
74.7	32,902.7	4813.2	8.1	6.8	0.84	0.519	0.516	0.634
84.9	105,031.5	14,854.8	18.3	7.1	0.39			
89.9	190,295.4	19,203.6	23.3	9.9	0.43			
96.1	330,272.3	26,704.3	29.5	12.4	0.42			
150.0^b^	3,344,358.5	79,269.8	83.4	42.2	0.51			

Range of ordinates for the water level	Volume of water between ordinates (VBO)	Surface area of the water reservoir on the higher ordinate (WSBO)	Water depth between ordinates (WDBO)	Average depth (AD_m_)	Individual reduction factor (IRF)	Average reduction factor (ARF_m_)		
						(ARF_m1_)	(ARF_m2_)	
m a.s.l.	m^3^	m^2^	m	m	–	–	–	
66.6–74.7	32,902.7	4813.2	8.1	6.8	0.84	0.763	0.752	
74.7–84.9	72,128.8	14,854.8	10.2	4.9	0.48			
84.9–89.9	85,263.9	19,203.6	5.0	4.4	0.89			
89.9–96.1	139,976.9	26,704.3	6.2	5.2	0.85			
96.1–150.0^b^	3,014,086.1	79,269.8	53.9	38.0	0.71			

^a^Water volume calculated from the 66.6 m a.s.l. ordinate as the start ordinate^14,32^.

^b^Prognosed value^14,32^.

The area of granite quarries situated in the town Strzelin is located on the watershed between the catchments of Ślęza and Oława Rivers, with a larger part in the catchment of the Ślęza River^93,94^. The analysed quarry lake Strzelin (shown in Fig. 2 as quarry lake 1) is drainless, and from the geographic point of view (according to the hydrological map of Poland^93,94^) is located in the catchment of the Oława River. However, it is partly supplied from the direct catchment (located in the catchment of Oława and Ślęza Rivers) and by transfer of water from the other pits (located in the catchment of the Ślęza River)^14,32^. As a result, quarry lake Strzelin was often assigned to the catchment of Ślęza.

The total volume of water retention in balance catchments (WR08 Bystrzyca and WR09 Nysa Kłodzka) and small-scale reservoir and pond retention in the catchments of the Oława and Ślęza Rivers was determined based on the inventory of the objects discussed above and the presented strategic and planning documentation related to water management^64,72,108^.

## Results and discussion

As a result of the inventory, it was found that the retention capacity of large reservoirs in the WR08 Bystrzyca balance catchment had a total volume of 16.19 hm^3^ and the small-scale pond and reservoir retention was 5.333 hm^3^ (reservoirs: 3.910 hm^3^, and ponds: 1.423 hm^3^)^64,72^. In the Ślęza catchment, the total volume of small-scale reservoir and pond retention was 0.809 hm^3^ (reservoirs: 0.552 hm^3^, ponds: 0.257 hm^3^), where the volume retained in closed quarry pits amounted to 0.018 hm^3^^64,72^. The inventory of balance catchment WR09 Nysa Kłodzka revealed a total retention volume in large reservoirs of 104.96 hm^3^ and a total volume of small-scale reservoir and pond retention of 4.122 hm^3^ (reservoirs: 2.039 hm^3^, ponds: 2.083 hm^3^)^64,72^. The total volume of small-scale reservoir and pond retention in the Oława catchment amounted to 2.782 hm^3^ (reservoirs: 1.871 hm^3^, ponds: 0.911 hm^3^). The volume retained in closed quarry pits accounted for 0.245 hm^3^ (including 9.6 thousand m^3^ in one quarry)^64,72,108^.

**Figure 4:**
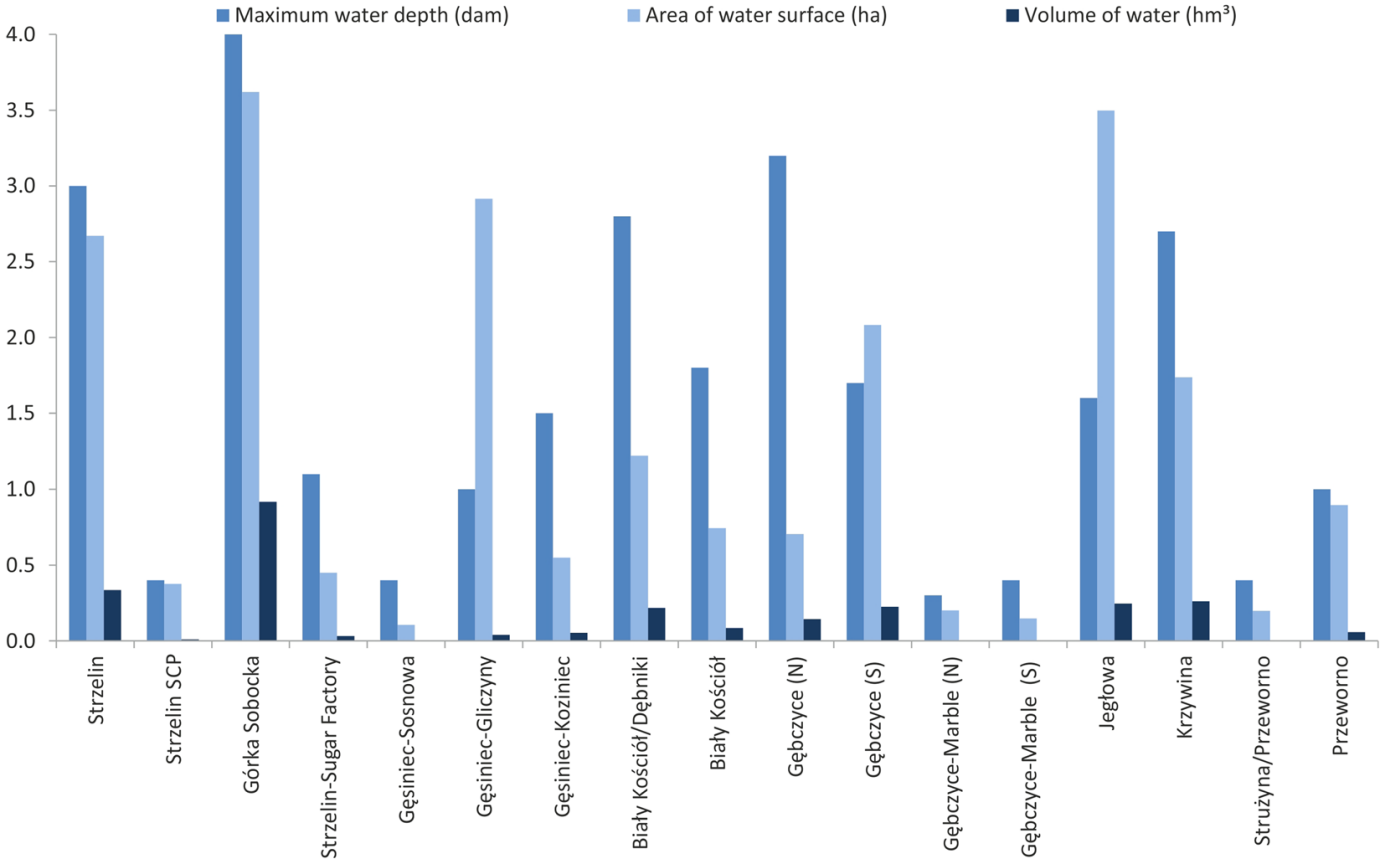
Water surface area, maximum depth of the analysed SQLs and volume of water retained in individual SQLs.

**Figure 5:**
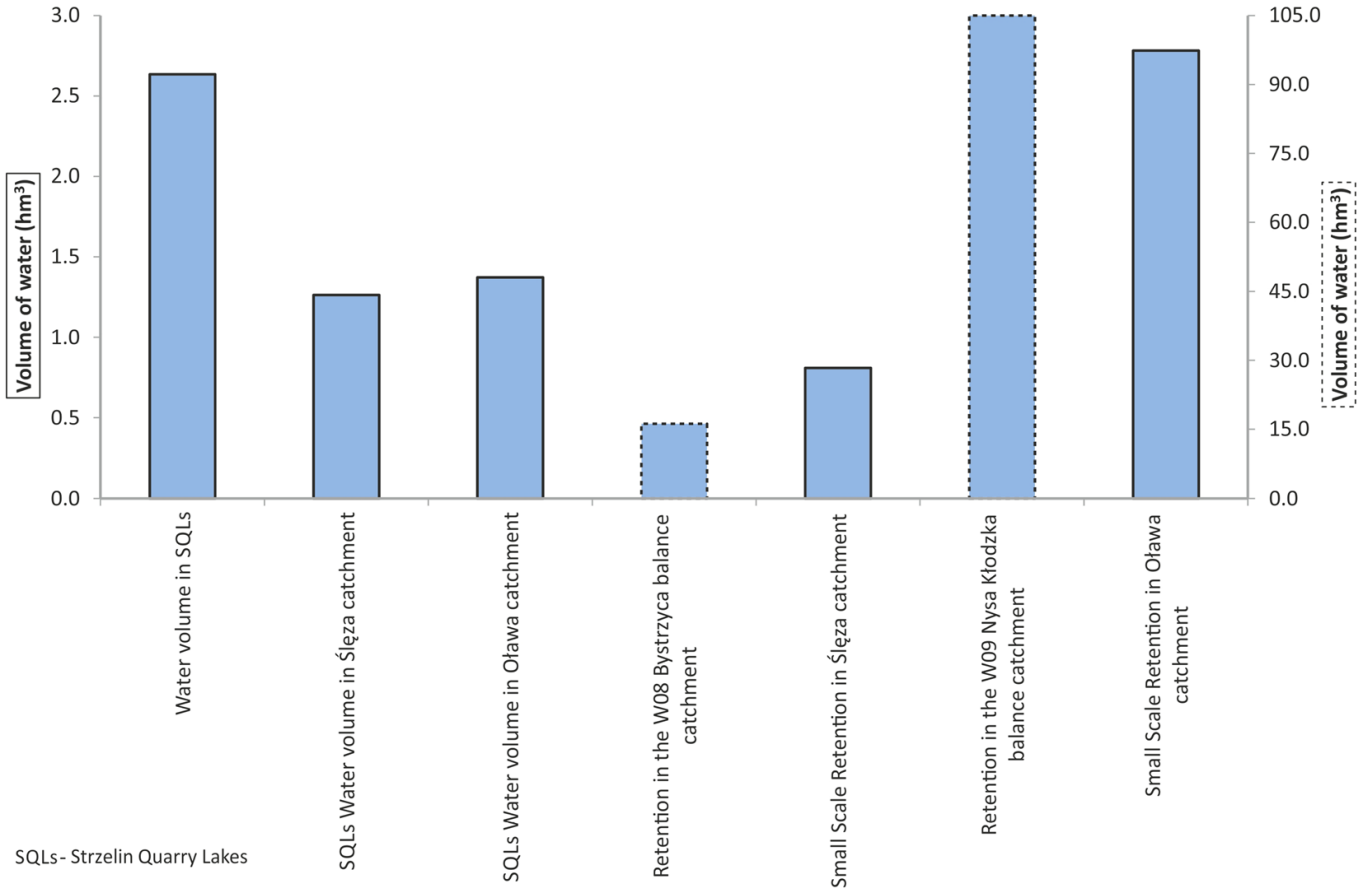
Volume of water retained in small-scale reservoir and pond retention and water retained in Strzelin Quarry Lakes (left scale—the water volume value for columns with solid border lines, right scale—the water volume value for columns with dotted border lines).

Seventeen quarry lakes were found in the area of Strzelin County, 6 of which were located in active quarries. Most of the Strzelin Quarry Lakes (14) are situated in the catchment of the Oława River, and only 3 of them are located in the catchment of Ślęza River (Fig. 2, Table 2). They are characterised by relatively small water surface areas, ranging from 0.11 to 3.62 ha (1.30 ha on the average), but their average maximum depth is 16.0 m (ranging from ~ 3 to ~ 40 m). The largest and deepest quarry lakes are Górka Sobocka (3.62 ha, 40 m) and Strzelin (2.67 ha, 30 m) (Table 2, Fig. 4). The volume of water retained in individual SQLs ranges from ~ 3 thousand m^3^ to 918 thousand m^3^ (Table 2, Fig. 4). The total volume of water retained in SQLs amounts to 2.635 hm^3^, of which 1.262 hm^3^ are retained in the catchment of the Ślęza River and 1.373 hm^3^ in the catchment of the Oława River (Table 2, Fig. 5). The largest amount of water is retained in quarry lake Górka Sobocka (0.918 hm^3^) and quarry lake Strzelin (0.335 hm^3^) (Table 2, Fig. 4). Both of them are located in the catchment of the Ślęza River, (Fig. 2) which belongs to the balance catchment WR08 Bystrzyca. One should remember that in active quarries, mining works are still conducted, which leads to an increase in the volume of the pit and its potential retention capacity. At the same time, some of the water from quarry lakes located in active quarries may be pumped out in order to exploit deeper parts of the deposit.

**Table 2:** Characteristics of Strzelin Quarry Lakes.

	Quarry lake	Type of resource mined	Water surface area (ha)	Maximum depth (m)	Water volume (thousand m^3)^	Catchment of river	Balance catchment
1	Strzelin^a^	Granite	2.67	30	334.90	Ślęza	WR08 Bystrzyca
2	Strzelin SCP^a^	Granite	0.38	4	9.53		
3	Górka Sobocka^a^	Granite	3.62	40	918.01		
4	Strzelin-Sugar Factory	Granite	0.45	11	31.38	Oława	WR09 Nysa Kłodzka
5	Gęsiniec-Sosnowa	Granite	0.11	4	2.68		
6	Gęsiniec-Gliczyny	Tonalite, Diorite, Granite	2.92	10	39.67		
7	Gęsiniec-Koziniec	Granite	0.55	15	52.25		
8	Biały Kościół/Dębniki	Granite	1.22	28	216.98		
9	Biały Kościół	Granite	0.74	18	84.92		
10	Gębczyce-North (N)^a^	Granite	0.70	32	142.91		
11	Gębczyce-South (S)^a^	Granite	2.08	17	224.51		
12	Gębczyce- Marble North (N)	Marble	0.20	3	3.81		
13	Gębczyce-Marble South (S)	Marble	0.15	4	3.76		
14	Jegłowa^a^	Quartzite and sericite shale	3.50	16	246.26		
15	Krzywina	Quartzite, quartzite and sericite slate	1.74	27	261.51		
16	Strużyna/Przeworno	Quartzite	0.20	4	5.12		
17	Przeworno	Marble	0.90	10	56.85		
		Average	1.30	16.2	∑ 2635.0		

^a^Situated in active quarries.

**Figure 6:**
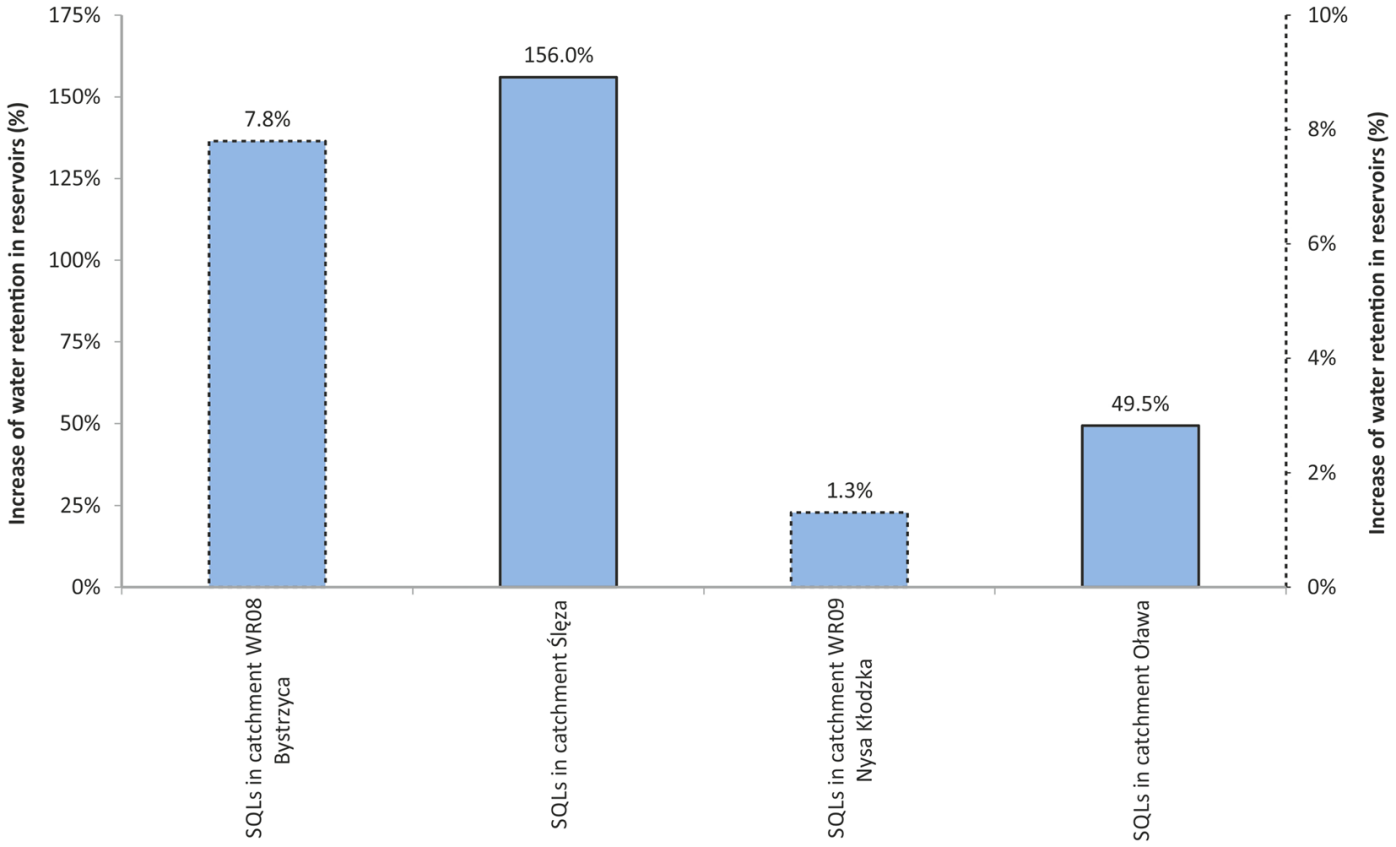
The influence of the volume of water retained in Strzelin Quarry Lakes on the increase in small-scale reservoir and pond retention in catchments [left scale (solid line)—the increase of water reservoirs retention in catchments, for columns with solid border lines; right scale (dotted line)—the increase of water reservoirs retention in catchments, for columns with dotted borders lines].

The comparison of the volume of water retained in SQLs with the volume retained in water reservoirs and ponds in the catchments of the Oława and Ślęza Rivers and the balance catchments WR08 Bystrzyca and WR09 Nysa Kłodzka (Fig. 5) revealed a significant increase in the volume of water retained as part of small-scale reservoir and pond retention. It should be noted that only some of the SQLs were included in the balance of small-scale reservoir and pond retention, while most of these objects was omitted^14,32,64,72^. Taking into account the volume of water retained in SQLs located in the Oława River catchment in the balance of water retained in reservoirs and ponds in this catchment (Fig. 5) resulted in an increase in the volume of water retained in the said catchment by approx. 49.5%, and in the balance catchment WR09 Nysa Kłodzka by approx. 1.3% (Fig. 6). On the other hand, considering the amount of water retained in SQLs located in the Ślęza River catchment (Fig. 5), led to an increase in the amount of water retained in the catchment by approx. 156%, and in the WR08 Bystrzyca balance catchment by approx. 7.8% (Fig. 6).

The volume of water retained in SQLs (Fig. 5) was not included in the balance of reservoir retention capacity in the catchments of the Oława and Ślęza Rivers. As a result, it is not taken into consideration in the water management plans, programmes and strategies, in particular those that concern water retention and distribution. This leads to the lack of reasonable proposals concerning the use of the water retained in SQLs. Currently, the availability of this water for the economy is quite poor and the water is used to a limited extent. In active quarries (No. 1–3, 10, 11, and 14) some of it is used in technological processes, for cutting rocks, washing aggregates, and in water curtains that reduce dust emission and the sprinklers of the loading shafts and transport roads. Some water is also discharged to the network of ditches and watercourses, periodically increasing their flow volume. The water from some SQLs (No. 11 and 14) is used regularly to maintain the water level in fish ponds and other water reservoirs. All the inactive SQLs (No. 4–9, 12, 13, and 15–17) are used for water recreation purposes, mainly swimming and amateur fishing, although only two of them (No. 5 and 17) were developed professionally, e.g. as a diving site (No. 17).

Most of the SQLs are located in agricultural or forest areas, but none of them are a source of water supply for agricultural or forest irrigation. Due to their location, the potential use of SQLs in water management of the analysed catchments will be mainly of local importance. However, taking the SQLs into account in the balance of reservoir water retention in the catchments of the Oława (1.373 hm^3^ (Fig. 5)) and Ślęza Rivers (1.262 hm^3^ (Fig. 5)) will enable to include the actions related to the development and renovation of the infrastructure for distributing water from SQLs to potential places of use in the water management plans, programmes and strategies. The low water levels, and even periodical drying of watercourses that persists in some of the rivers in Strzelin County, as well as the agricultural nature of the region will, in fact, point to agriculture as the main direction of use of waters from SQLs. Considering the limited resources of surface waters in Strzelin County, the water retained in SQLs (which has been used to a limited extent so far) may contribute to improving the availability of surface waters in the county, for example for agricultural purposes. Apart from that, focusing on the potential of the water retention possibilities offered by quarries should translate into the appropriate water management of quarries that are being shut down. This may be quite important in the regional aspect, as Lower Silesia is home to dozens of active quarries as well as hundreds of inactive quarries, some of which are flooded.

The assessment of water quality in selected SQLs was the subject of other publications that discussed, among others, the assessment of the content of biogenic substances^54^ and the suitability of waters from flooded mining pits for irrigation in agriculture^52,53^. The selected results and conclusions of the water quality assessment in selected SQLs are quoted below.

The results of the assessment of the content of biogenic substances^54^ in the waters of SQLs No. 7, 8, 9, 15, and 17 (Fig. 2) demonstrate that the average total N content fell into the range of 1.10–3.50 mgN dm^−3^ and total P in the range of 0.39–1.08 mgP dm^−3^. The high concentration of total P in all five analysed SQLs (> 0.1 mgP dm^−3^) and the content of total N (> 1.5 mgN dm^−3^) in SQLs 8 and 9 points to the eutrophication of the waters of the analysed SQLs, which manifests itself in algae blooms. No risk of contamination with nitrates from agricultural sources was found, as the maximum concentration of NO_3 _in the analysed SQLs was 1.06 mgNO_3_^−^  dm^−3^, and the threat caused by nitrates from agricultural sources occurs at levels above 40 mg NO_3_^−^ dm^–3^^109^. The assessment of the suitability of the waters from the analysed SQLs, as a source of potable water supply for residents revealed that SQLs No. 7, 15, and 17 were classified in the highest category A1, SQL No. 8 in the A2 category (due to increased pH), while the water from SQL No. 9 was unsuitable for consumption by humans (exceeded threshold values of pH)^110^. Due to low oxygenation and excessive concentration of phosphates in the waters of SQLs No. 7, 8, 9, 15, and 17 (Fig. 2) the waters did not meet the requirements for fish life in natural conditions^111^.

The assessment of the suitability of the waters from SQLs No. 3, 6, 8, and 14, and 17 (Fig. 2) for agricultural irrigation^52,53^ was based on the guidelines of the FAO and the Polish Standard PN-84/C-04635^112–114^. The average values of water quality indicators fell into the ranges: electrical conductivity of water (ECw) 0.163–0.681 mS cm^−1^; Sodium Adsorption Ratio (SAR) 0.35–1.96; Total Dissolved Solids (TDS) 117.1–396.0 mg dm^−3^; water pH 7.1–9.0; BOD_5_ 1.32–2.04 mgO_2_ dm^−3^. Average concentration of ions for specific SQLs, they fell into the ranges: nitrates 0.30–033.48 mgNO_3_^−^ dm^−3^; sulphates 156.32–233.48 mgSO_4_^2−^ dm^−3^; chlorides 15.20–56.92 mgCl^−^ dm^−3^; sodium 6.98–65.72 mgNa^+^ dm^−3^; calcium 21.44–60.90 mgCa^2+^ dm^−3^; magnesium 4.00–16.48 mgMg^2+^ dm^−3^; manganese 0.008–0.090 mgMn dm^−3^; iron 0.002–0.078 mgFe dm^−3^; zinc 11.32–21.02 µgZn dm^−3^; copper 18.16–24.04 µgCu dm^−3^; cadmium 0.50–1.48 µgCd dm^−3^; lead 2.52–3.62 µgPb dm^−3^; chromium 2.06–6.24 µg Cr dm^−3^; nickel 0.46–10.30 µgNi dm^−3^. Most of indicators for the waters of the analysed SQLs (No. 3, 6, 8, and 14, and 17) met the requirements foreseen in the guidelines on the use of water for irrigation^112–114^. Due to high pH value, water from SQL No. 3 did not meet the requirements provided in PN-84/C-04635. Due to increased nitrate content in SQL No. 3, and excessive sodium concentration and the value of ECw in SQL No. 17, water from these quarry lakes should be subject to slight to moderate restriction on use for irrigation. According to the guidelines of FAO, the assessment of sodium hazard of irrigation water based on SAR and ECw suggests severe restrictions in using water from SQL No. 4 for irrigation and slight to moderate restriction in using the water from SQLs No. 3, 6, 8, and 17 for irrigation. Due to high natrium content, severe restrictions on using water from SQLs No. 3, 6, 8, and 14, and 17 for surface irrigation should be applied. As a result of high pH, water from SQL No. 3 is subject to high restrictions on use for drip irrigation and water from SQLs No. 6, 8, and 14, and 17 should be subject to moderate restrictions on use for drip irrigation. As far as the concentration of heavy metals is concerned, the water from the analysed SQLs (No. 3, 6, 8, and 14, and 17) met the requirements that allow it to be used for agricultural irrigation, in particular sprinkler irrigation.

The poor result of the assessment of the chemical condition in SQLs No. 7, 8, 9, 15, and 17, which resulted mainly from the exceeded threshold values for total P, might seem problematic. In quarry lakes, the sources of this element may be the organic matter supplied to the reservoir from belts of trees and bushes and from the forests that surround the SQLs. It may also be released from bottom sediments, and in SQLs No. 8 and 9 it may likely originate from the pressure from municipal and agricultural wastewater. However, the concentrations of selected water quality indicators in SQLs No. 3, 6, 8, 9, and 14, 15, and 17 were similar to the concentrations found in natural lakes not subjected to load, and decidedly lower than the concentrations found in most mine lakes, in particular Acid Mine Lakes and mine lakes in former metal ore excavation sites^52–54^. One may suppose that the water from SQLs should not have a significant negative influence on the water quality in the analysed catchments, however it is recommended to continue and expand the scope of research on water quality in SQLs.

## Summary and conclusion

Quarry lakes, as post-mining objects, are very often not included in balances of retention capacity of water reservoirs that are located in the catchments of Polish rivers, which is demonstrated by the analysis conducted for the catchments of the Oława and Ślęza Rivers^64,72,108^. Including the volume of water retained in Strzelin Quarry Lakes in the balances of retention capacity of water reservoirs situated in the catchments of the Oława and Ślęza Rivers resulted in a significant increase in the water resources in retention reservoirs in the Oława catchment (by approx. 50%) and in the Ślęza catchment (by approx. 156%). This confirmed the results of previous study on the influence of the volume of water retained in a single granite quarry in Strzelin^14,32^, on the balance of the retention capacity of water reservoirs, thus being part of the national plan for counteracting the effects of drought^63^. This might improve the water balance in the catchment, especially in the part concerning of water retention^3,6,7,68,70^ and mitigate the consequences of water deficits. The water retained in quarry lakes may be used for economic and/or environmental purposes. In the event of very low water levels in small natural water courses (e.g. those that are valuable from the natural point of view), water from quarry lakes may be used to supply the water course periodically e.g. to maintain the environmental flow. However, the water from the quarry lake must be of appropriate quality, so as not to deteriorate the quality of water in the watercourse, and not to endanger its ecosystem. Water from quarry lakes may also constitute a source of water supply for industrial plants, e.g. those that use flowing waters, when the flow rate in the water course falls to a level equal or lower than the environmental flow. In the event of long-term drought, it may also be a source of supplying potable water to residents. During agricultural drought, water from quarry lakes may be used as a source of water for irrigation. It may also serve as a source of water supply for breeding ponds, e.g. to fill the pond or to maintain the water inflow, especially when the water levels in watercourses are low and the losses caused by evaporation and leaks from the pond. This becomes particulay important in agricultural areas, such as Strzelin County^14,32^, especially during droughts and periodical drying of watercourses in the analysed catchments^115^. It is important that the water from quarry lakes should meet the quality requirements for the purpose, for which it will be used. For example, water from selected SQLs meets the requirements for the supply of potable water and of water for agricultural irrigation^52–54^.

Thus, if the hydrological, geohydrological, and geomorphological conditions are positive, it seems natural to establish retention reservoirs in closed and reclaimed quarries. This will reduce the construction costs of water reservoirs (the excavation pit will be transformed into the basin of the reservoir) and improve the effectiveness of the realisation of tasks foreseen as part of adapting such areas as agriculture, water management, forestry, and industry to climate changes. Due to that, it is worth including the appropriate provisions that would prioritise the water direction of reclamation of post-mining areas in the relevant planning and strategic documents related to environmental protection, water management, adapting to climate changes, and preventing the consequences of droughts and floods.

The conducted research allowed the Author to formulate the following conclusions:

1. Taking the volume of water retained in Strzelin Quarry Lakes [2.635 hm^3^, 1.262 hm^3^ in the catchment of the Ślęza River (WR08) and 1.373 hm^3^ in the catchment of the Oława River (WR09)] into account in the balance of the capacity of retention reservoirs and ponds in the catchments of the Ślęza and Oława Rivers will result in a significant increase in the balance of the retention capacity of water reservoirs: by 156% on the catchment of the Ślęza River and by 49.5% in the catchment of the Oława River, as well as in the balance catchments: Bystrzyca (WR08) by 7.8% and Nysa Kłodzka (WR09) by 1.3%.
2. Due to the mainly agricultural nature of Strzelin County, it seems that agriculture should be the main user of the waters retained in the SQLs. Adding SQLs to the register of water reservoirs and to the balance of the retention capacity of water reservoirs should result in actions aimed at the reconstruction and development of systems that enable the distribution of water from SQLs to recipients. It should also lead to the development of water management programmes, including counteracting the effects of droughts and adapting to climate changes. This should result in improved availability of water for potential recipients, in particular in agriculture.
3. The concentrations of selected water quality indicators in the SQLs were similar to those noted in natural lakes and water reservoirs not subjected to loads, and lower than concentrations found in mine lakes. The parameters of water in SQLs showed that it might potentially be useful for agricultural irrigation (in particular sprinkler irrigation) and for supplying potable water to residents, even if the total P concentrations were slightly increased, pointing to the eutrophication of the SQLs.

## Data Availability

All data generated during this study are included in this published article. The data that support the findings of this study are available from Chief Land Surveyor of Country, Warsaw, Masovian Voivodeship, Poland (licence no. DIO.7211.204.2019_PL_N, licence no. DIO.7211.160.2018_PL_N) and Marshal of the Lower Silesian Voivodeship, Wrocław, Lower Silesian Voivodeship, Poland (license No. MGW.I.7522.524.2016_02_N, licence no. MGW-I.7552.26.2019_02_N) but restrictions apply to the availability of these data, which were used under license for the current study, and so are not publicly available. Data are however available from the authors upon reasonable request and with permission of Chief Land Surveyor of Country (Poland) and/or Marshal of the Lower Silesian Voivodeship (Poland).
